# An Image-Guided Percutaneous Core Needle Biopsy for Accurate Assessment of Musculoskeletal Tumors: A Targeted Diagnosis

**DOI:** 10.7759/cureus.60757

**Published:** 2024-05-21

**Authors:** Bodla Arvind Kumar, Shravan Peddamadyam, Vamsi Krishna, Nageswara Rao Kancherla, Kotesh N Nayak, Nagesh Cherukuri, Shantveer G Uppin

**Affiliations:** 1 Department of Orthopaedics, Nizam’s Institute of Medical Sciences, Hyderabad, IND; 2 Department of Orthopaedic Surgery, Nizam’s Institute of Medical Sciences, Hyderabad, IND; 3 Department of Pathology and Laboratory Medicine, Nizam’s Institute of Medical Sciences, Hyderabad, IND

**Keywords:** bone tumor and limb salvage, diagnostic accuracy, image guidance, percutaneous core needle biopsy, musculoskeletal tumor

## Abstract

Background

Accurate diagnosis of musculoskeletal tumors is essential for guiding appropriate treatment strategies. Percutaneous core needle biopsy (PCNB) is increasingly recognized as a valuable method for obtaining tissue samples for histopathological examination. This study aims to evaluate the diagnostic accuracy and clinical utility of PCNB in diagnosing musculoskeletal tumors.

Methodology

A total of 152 cases suspected of musculoskeletal tumors underwent PCNB at our tertiary care center between 2020 and 2023. Pre-biopsy evaluation included comprehensive clinical assessment and imaging studies. Core biopsies were performed under image guidance, with specimens sent for histopathological examination and culture sensitivity analysis. Diagnostic yield, accuracy, and performance metrics of PCNB were assessed.

Results

PCNB demonstrated a diagnostic yield of 93.4%. However, in cases where initial biopsies were inconclusive, repeat core biopsy or open biopsy provided the necessary diagnostic clarity. PCNB demonstrated a remarkable diagnostic accuracy of 97.9%, with a specificity and positive predictive value of 100%. There were no post-biopsy complications and no instances of local recurrence from the biopsy tract.

Conclusions

PCNB can be a reliable method for diagnosing musculoskeletal tumors, offering high diagnostic accuracy and minimal complications. The utilization of image guidance enhances precision and reduces the risk of complications. PCNB proves effective in diagnosing both primary tumors and bone infections, facilitating timely and appropriate treatment strategies in orthopedic oncology.

## Introduction

Accurate diagnosis is crucial for effectively managing musculoskeletal tumors, and biopsy plays a pivotal role in discerning bone and soft tissue tumor types [1,2]. The primary objective of biopsy is to procure diagnostic tissue while minimizing associated risks such as morbidity and potential tumor spread and to ensure that future treatments are not compromised. It is prudent to approach all lesions as potentially malignant until proven otherwise, necessitating that biopsy be deferred until imaging studies are complete. This approach allows for comprehensive assessment, correlating radiographic findings with histological examination. Staging of the lesion further aids in determining the precise anatomical approach to the tumor and delineating the specific area of the tumor that signifies the underlying pathology [2].

Open biopsy has long been regarded as the gold standard for procuring tissue samples for histological diagnosis with a reported diagnostic accuracy of approximately 98%. However, this method is not without its drawbacks, as it is associated with various complications, including tumor spillage, hematoma, subcutaneous hemorrhage, and infection. Moreover, the procedure is costly, requires hospitalization, and necessitates anesthesia administration [3]. Consequently, the complication rate of open biopsy has been documented to be approximately 16%, posing potential obstacles to subsequent treatment procedures and limb salvage efforts, particularly when incisions are not appropriately marked [3,4].

On the other hand, percutaneous core needle biopsy (PCNB) has been acclaimed for its lower complication rates (0-2%), along with reduced morbidity, cost, and time requirements [5]. This procedure can be conveniently performed under local anesthesia in an outpatient setting, with a reported diagnostic accuracy ranging from 76% to 99%. Thus, it is considered safe and effective for the diagnosis of musculoskeletal tumors [6]. Utilizing image guidance such as fluoroscopy and CT guidance enhances precision, minimizes the risk of injury to major neurovascular structures, and confirms the site of tissue acquisition [7]. In this study, we assessed the utility of core needle biopsy in the diagnosis of musculoskeletal tumors.

## Materials and methods

We conducted a prospective observational study, enrolling 152 subjects who underwent PCNB at Nizam’s Institute of Medical Sciences between 2020 and 2023.

Pre-biopsy evaluation and planning

**Figure 1:**
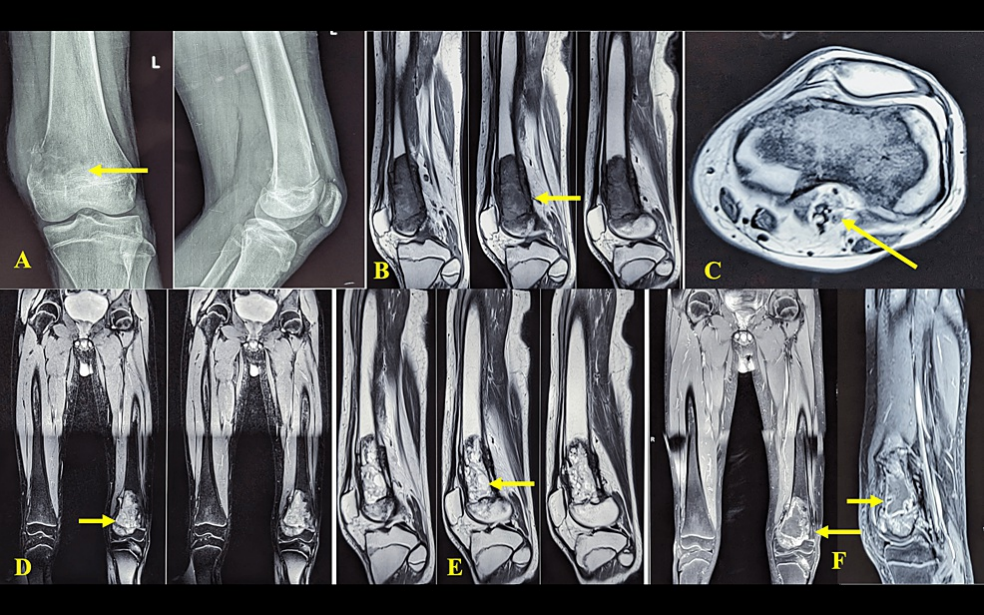
Pre-biopsy imaging evaluation: X-ray and MRI (plain and contrast). A: Plain X-ray of the left knee showing a lesion in the distal femur with a cortical break on the medial aspect. B: T1 sagittal section showing the lesion. C: T1 axial section showing the lesion and its relationship to the neurovascular bundle. D: T2 coronal section of the whole thigh including hip joints to rule out skip metastasis. E: T2 sagittal section showing the lesion. F: Contrast MRI images showing lesions in both coronal and sagittal sections.

**Figure 2:**
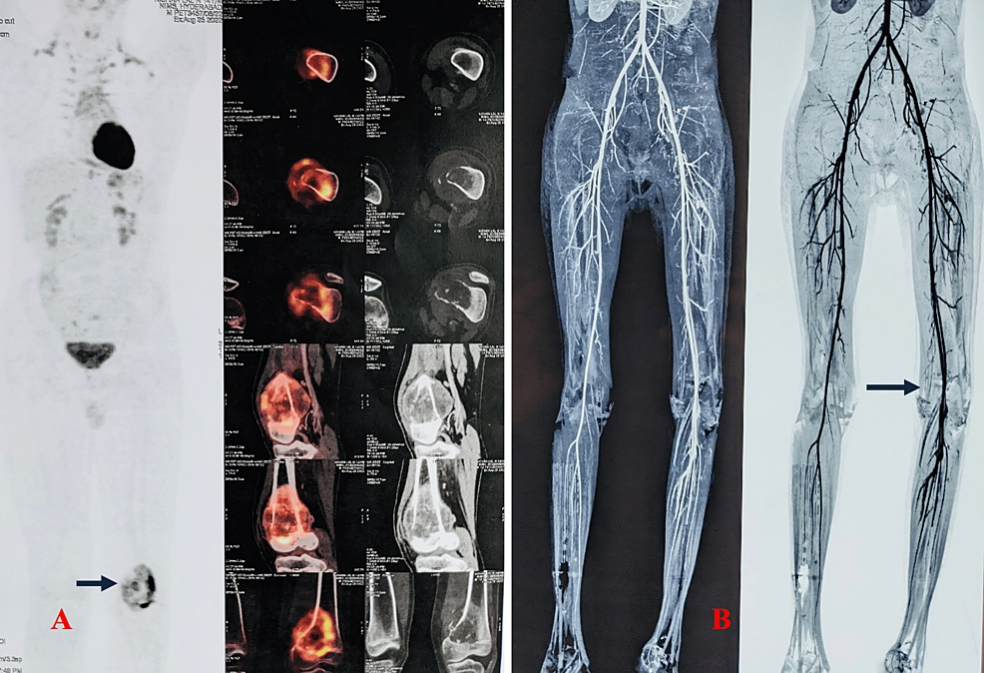
Pre-biopsy imaging evaluation: PET-CT and CT angiography. A: Whole-body PET-CT to rule out metastasis. B: CT angiography of the involved limb. PET-CT = positron emission tomography-computed tomography

The procedure involved a meticulous approach that began by obtaining a comprehensive patient history and conducting a thorough clinical examination. Subsequently, a series of imaging modalities were employed to provide a detailed assessment of the lesion. These included plain radiographs with scales and MRI of the involved region, encompassing both plain and contrast sequences. These imaging studies aim to ascertain various aspects, such as the extent of the tumor, involvement of soft tissues, extension into adjacent joints, and the relationship with neurovascular structures. Additionally, a CT angiography of the limb was performed to evaluate the vascularity of the tumor and identify the feeder vessels supplying it. In cases where malignancy was suspected, positron emission tomography-computed tomography (PET-CT) was also conducted to assess for any signs of metastasis. The pre-biopsy imaging evaluation is shown in Figure 1 and Figure 2.

These imaging findings were interpreted meticulously by experienced orthopedic oncologists in collaboration with musculoskeletal radiologists to delineate a precise pathway for biopsy acquisition, ensuring that the resulting scar was excised during definitive surgery, and to identify neurovascular structures to minimize the risk of contamination and damage during the biopsy procedure.

Core biopsy procedure

**Figure 3:**
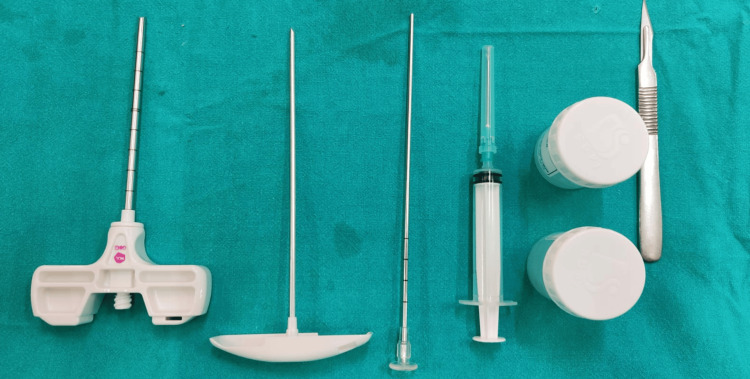
Armamentarium for core biopsy. The figure shows from left to right, the outer cannula, inner trocar, stylet, 10 cc syringe, sterile containers, No. 11 blade, and handle.

For both bone and soft tissue biopsies, we utilized an 8-gauge Jamshidi needle (Figure 3).

Before commencing the procedure, a comprehensive assessment of the patient’s coagulation profile and serology was conducted, with any identified abnormalities corrected. All biopsies were meticulously performed under sterile conditions in the operating room by a trained orthopedic oncologist who also undertook the final definitive procedure, and the targeted area was prepared and draped in a sterile manner. Subsequently, 2% lignocaine was injected for local anesthesia. A No. 11 blade was used to create a precise skin incision. The core biopsy needle was carefully advanced into the lesion and its placement was confirmed under fluoroscopic guidance. If the lesion was not suitable for PCNB under fluoroscopic guidance due to the presence of nearby neurovascular structures or if the lesion was deeply seated, such as in the pelvis, either ultrasonography (USG) or CT guidance was utilized.

**Figure 4:**
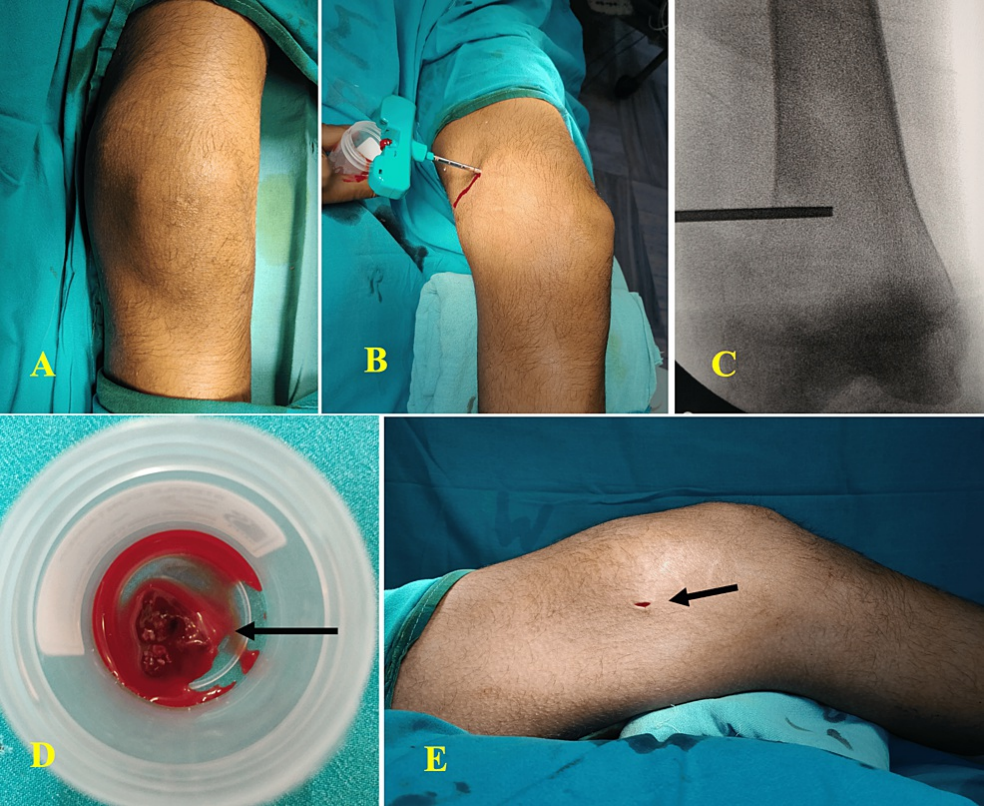
Core biopsy procedure. A: Sterile draping of the involved site. B: Core biopsy needle placement. C: Fluoroscopic guidance. D: Cores collected in a sterile container. E: Core biopsy wound at the end of the procedure.

Multiple cores (2-3) were obtained from the same incision by directing the needle in different orientations within the lesion. The core biopsy procedure is depicted in Figure 4.

Following the biopsy, a sterile compression dressing was applied to the site. The obtained tissue specimens were promptly placed in a sterile container and immediately sent for both histopathological examination and culture sensitivity analysis. This meticulous approach ensured the integrity of the samples and facilitated accurate diagnosis and appropriate treatment planning. The biopsy cores underwent fixation in formalin and standard processing for histological assessment, utilizing hematoxylin and eosin staining. Expert pathologists with specialization in musculoskeletal pathology meticulously reviewed the slides. Special stains were employed when necessary to aid in achieving specific diagnoses.

Statistical analysis

Data Analysis was performed using SPSS software (IBM Corp., Armonk, NY, USA). The diagnosis obtained from the core needle biopsy was compared with the final diagnosis determined by definitive surgery. The diagnostic yield, sensitivity, specificity, positive predictive value (PPV), and negative predictive value (NPV) of core needle biopsy were calculated.

## Results

Demographic and procedural characteristics

The study enrolled 152 cases of suspected musculoskeletal tumors, consisting of 89 males and 63 females, with a mean age of 26.5 years (range = 5-68 years). PCNB under fluoroscopic guidance (C-arm) was the predominant procedure and was conducted in 142 cases. Additionally, six patients underwent biopsy under CT guidance due to pelvic lesion location, while four patients underwent core needle biopsy under USG guidance. Local anesthesia was administered to 116 patients, whereas sedation was used in 30 patients aged less than 15 years. Patients who underwent CT-guided biopsy received spinal anesthesia (n = 6). The sociodemographic characteristics of the study cohort are depicted in Table 1.

**Table 1:** Sociodemographic characteristics of the study population.

Variables	Categories	Frequency (%)
Age, years	<20	62 (40.8)
	21–40	69 (45.4)
	>40	21 (13.8)
	Mean age = 26.5 years	
Gender	Male	89 (58.5)
	Female	63 (41.5)
Biopsy guidance	Fluoroscopic	142 (93.42)
	CT	6 (3.95)
	Ultrasound	4 (2.63)
Type of anesthesia	Local	116 (76.3)
	Sedation	30 (19.7)
	Spinal	6 (4.0)
Core biopsy diagnosis	Benign	80 (58.4)
	Malignant	57 (41.6)
Final biopsy diagnosis	Benign	85 (59.44)
	Malignant	58 (40.56)

Lesion characteristics

Most lesions were located around the knee (n = 85, 56.29%), with the distal femur and proximal tibia being the most common sites. The lesion distribution across different anatomical regions was as follows: upper extremity - 33, lower extremity - 112, pelvis - 6, and spine - 1. The distribution of tumors according to the site is depicted in Table 2 and Table 3.

**Table 2:** Lesion localization patterns.

Location of the lesion	Frequency (%)
Upper extremity	33 (21.85)
Lower extremity	112 (74.17)
Pelvis	5 (3.31)
Spine	1 (0.66)

**Table 3:** Anatomical distribution of lesions. Others include the clavicle, ulna, radius (shaft), metacarpal, metatarsal, calcaneum, and talus.

Anatomical location	Frequency (%)
Distal femur	46 (29.11)
Proximal tibia	38 (24.05)
Proximal humerus	13 (8.23)
Proximal femur	12 (7.59)
Distal radius	9 (5.70)
Distal tibia	5 (3.16)
Pelvis	6 (3.57)
Others	22 (13.14)

Diagnostic yield and biopsy outcomes

In our study, of the 152 biopsied specimens, diagnoses were not identified in 10 cases due to inadequate biopsy material on the initial core biopsy, resulting in a diagnostic yield or adequacy rate of 93.4% (142/152). Among the remaining 142 specimens that were subjected to core biopsy, giant cell tumors (n = 52, 36.6%) were the most common diagnoses in the benign category, whereas osteosarcoma (n = 38, 26.7%) predominated in the malignant category. The diagnosis of tumors obtained by the core biopsy is tabulated in Table 4.

**Table 4:** Tumors diagnosed by core biopsy. Variants include secondary components such as aneurysmal bone cysts or benign fibrohistiocytic changes. Cystic lesions include aneurysmal bone cysts and solitary bone cysts. Other lesions include desmoplastic fibroma, low-grade mesenchymal neoplasm, low-grade chondral neoplasm probably enchondroma, and fibrous dysplasia.

Tumor category	Type of tumor	Frequency (%)
Benign	Giant cell tumor and its variants	52 (36.61)
	Chondroblastoma and its variants	12 (8.45)
	Cystic lesions	6 (4.22)
	Osteoclast giant cell-rich lesions	3 (2.11)
	Chondromyxoid fibroma	2 (1.40)
	Others	5 (3.52)
Malignant	Osteosarcoma	38 (26.7)
	Chondrosarcoma	12 (8.45)
	Ewing’s sarcoma	5 (3.52)
	Leiomyosarcoma	2 (1.40)

For cases in which the lesion was not identified on the initial core biopsy (10 cases), repeat core biopsy was performed in seven cases, and open biopsy was conducted in the remaining three cases. All seven cases subjected to repeat core biopsy yielded a diagnosis, with the majority identified as aneurysmal bone cysts (five cases), one as a giant cell tumor with an aneurysmal bone cyst component, and one as an enchondroma. The three patients who underwent open biopsy were diagnosed with a tubercular infection of the distal tibia, epithelial hemangioma of the pelvis, and epithelioid hemangioendothelioma of the proximal humerus. One patient, initially diagnosed with a giant cell tumor of the distal tibia via core biopsy, underwent tumor resection with fibula transposition and ankle arthrodesis. However, the final biopsy revealed a giant cell tumor with sarcomatous changes, which were not initially reported because of inadequate tissue sample representation (Table 5).

**Table 5:** Discrepancies between core needle biopsy and final histological diagnosis.

Category	Frequency, N	Core needle biopsy diagnosis	Open biopsy diagnosis
Category 1: Benign on core biopsy but turns out to be malignant on open biopsy	1	Giant cell tumor of the bone	Giant cell tumor of the bone with secondary sarcomatous changes
Category 2: Lesion not identified on core biopsy but identified on open biopsy	2	No lesion identified	Epitheliod hemangioma (intermediate category)
		No lesion identified	Epitheliod hemangioendothelioma
Category 3: Benign on core biopsy as well as open biopsy but different diagnosis	2	Low-grade mesenchymal tumor	Phosphaturic mesenchymal tumor
		Desmoplastic fibroma	Fibrocartilagenous mesenchymoma

Five cases were diagnosed with osteomyelitis based on core biopsy findings, which were further supported by positive culture reports. These patients underwent treatment involving pus drainage and intravenous antibiotics, resulting in a successful recovery.

Performance metrics of percutaneous core needle biopsy

The diagnostic accuracy of PCNB in diagnosing malignant tumors was approximately 96.6%, whereas that of benign tumors was approximately 98.8%. Core biopsy could distinguish between benign and malignant tumors in 99% of the cases. The overall sensitivity of core needle biopsy was 97.9%, with a specificity of 100%, a PPV of 100%, and an NPV of 75% (Table 6).

**Table 6:** Diagnostic accuracy of core needle biopsy.

Variable	Frequency	Percentage
True positives	145	96.6
True negatives	6	4
False positives	0	0
False negatives	2	1.3

No major complications were identified in the post-procedure period.

## Discussion

In the realm of orthopedic oncology, obtaining a histopathological diagnosis of bone tumors is indispensable for guiding appropriate treatment strategies. This diagnostic endeavor necessitates a collaborative effort between orthopedic surgeons responsible for procuring biopsy specimens from the lesion and experienced pathologists tasked with histological interpretation. Historically, open biopsy has been the gold-standard technique for acquiring tissue samples, but it has significant drawbacks, notably a heightened risk of tumor cell contamination of the surrounding tissues [8]. Studies, such as that by Mankin et al. [9], have underscored the repercussions of suboptimal biopsy techniques, with amputation necessitated in a notable percentage of cases (4.5%). However, contemporary orthopedic oncology has witnessed a paradigm shift toward limb salvage procedures owing to advancements in chemotherapy, surgical techniques, and global accessibility to prosthetic options. In this context, PCNB has emerged as the preferred method, offering enhanced precision while minimizing the risk of complications associated with open biopsy.

Cannon [10] highlighted that 38% of patients in their study experienced local recurrence after open biopsy unless the biopsy site underwent excision. Notably, apart from the single case of osteosarcoma documented by Davies et al. [11], there have been no reports of tumor recurrence attributed to needle biopsy. Consistent with this trend, no instances of local recurrence originating from the biopsy needle tract were observed in our study. Our adherence to meticulous pre-biopsy and preoperative planning, coupled with the principle of excising the biopsy tract during definitive surgery, underscores the paramount importance of surgical involvement in the biopsy process. By ensuring that the surgeon, an integral part of the definitive surgical team, performs the biopsy, we aimed to minimize the risk of local recurrence and optimize patient outcomes. While open biopsy boasts a diagnostic accuracy of up to 96%, it is accompanied by a complication rate of approximately 16% [12]. Conversely, core biopsy is a safer alternative, with complication rates of less than 1% [5]. In our study, no complications were observed in patients who underwent PCNB. To manage any discomfort experienced in the post-procedure period, analgesics were prescribed in the recovery room.

Core needle biopsy offers a unique advantage in examining tumor architecture, thus facilitating precise diagnoses. Furthermore, the samples obtained through core biopsy are amenable to specialized testing and staining techniques, thereby enhancing diagnostic accuracy [13]. In our study, we employed specific stains tailored to different tumor types. For instance, we utilized h3.3 G34W stain for giant cell tumor [14], h3.3k36m stain for chondroblastoma [15], and satb2 and nkx2.2 stains for osteosarcoma and Ewings sarcoma. These specialized stains, meticulously selected by our pathologist, contributed to the accurate diagnosis of various lesions. In our study, the diagnostic accuracy of core biopsy in diagnosing malignant tumors was approximately 96.6%, whereas that of benign tumors was approximately 98.8%. Out of the 87 samples diagnosed as benign bone tumors on final histopathological examination, 79 (90.8%) cases were accurately diagnosed upon initial core biopsy. However, the remaining eight patients required repeat core biopsy to establish the diagnosis. This variance can be attributed to the characteristics of cystic lesions, such as aneurysmal bone cysts or simple bone cysts, which are fluid-filled, either with blood or fluid. This fluid-filled nature poses challenges for orthopedic specialists in obtaining diagnostic tissue for histopathological examination. Additionally, to achieve a definitive diagnosis of cystic bone lesions, scraping of the cyst wall alongside the fluid is often necessary [16].

Our study utilized various imaging modalities, including ultrasound, CT, and fluoroscopy, to accurately guide the needle to the target site during the core needle biopsy procedure. Hau et al. [17] reported that CT-guided biopsy is superior to conventional needle biopsy when the lesion is deep-seated, located in the pelvis or vertebrae, or very closely related to neurovascular structures. In our study, the majority of cases (93.4%) underwent PCNB with fluoroscopy (C-arm), while a smaller percentage was guided by a CT scan (3.94%) or ultrasound (2.63%) as the lesion was either located in the pelvis or near a neurovascular structure. These imaging techniques not only aid in precise lesion localization but also enhance diagnostic accuracy, mitigate the risk of injury to surrounding neurovascular structures, and minimize the potential for contamination of adjacent tissues by tumor cells [17].

In a singular instance, histological determination of malignancy proved elusive in the case of a giant cell tumor of the bone, initially reported as benign. However, upon surgical excision, secondary osteosarcomatous transformation was observed. Another challenging diagnosis involved epithelioid hemangioendothelioma, an aggressive tumor that eluded detection on initial core biopsy. Given the histological heterogeneity inherent in such tumors and the constrained sample size obtainable through needle biopsy, there is a risk of overlooking the most representative areas. To mitigate such diagnostic discrepancies, acquiring multiple samples from varying depths within the lesions may reduce the likelihood of misdiagnosis [4].

Several dedicated series examining outpatient core needle biopsies have reported diagnostic accuracies ranging from 84% to 98% [18-22]. In our series, the diagnostic accuracy of PCNB was at 97.9%, with a specificity of 100%, a PPV of 100%, and an NPV of 75 %, consistent with the findings of previous studies. Table 7 juxtaposes our study’s diagnostic accuracy of core biopsy in diagnosing musculoskeletal tumors with existing literature.

**Table 7:** Comparing our study against the existing literature.

Author	Year	Number of cases (n)	Adequacy rate/Diagnostic yield (%)	Diagnostic accuracy/Sensitivity (%)
Mitsuyoshi et al. [4]	2006	163	88%	97%
Puri et al. [18]	2006	136	79.41%	95.37%
Joshi et al. [19]	2013	59	92%	92.85%
Crenn et al. [1]	2021	196	84.7%	91.7%
Our study	2024	152	93.4%	97.9%

## Conclusions

PCNB has emerged as a dependable approach for acquiring representative tissue samples crucial for the histopathological examination of musculoskeletal tumors. This outpatient procedure is not only cost-effective but also straightforward to execute, offering precision with minimal complication rates. Its efficacy diminishes the necessity of initial open biopsy procedures, thus alleviating patient morbidity. In instances where the initial core biopsy fails to provide a diagnosis, subsequent core biopsies or open biopsy options remain viable with reduced patient risk. The incorporation of fluoroscopy, CT guidance, or USG guidance ensures precise needle tip placement within the lesion, thereby mitigating the occurrence of false-negative outcomes and minimizing the potential for damaging adjacent neurovascular structures. It is imperative to exercise caution, particularly when confronting cystic bone lesions such as aneurysmal bone cysts, where the initial diagnosis may yield inconclusive results due to inadequate sampling. In such scenarios, collaborative consideration of clinical and radiological parameters is essential, potentially warranting repeat biopsies with cyst wall scraping to yield diagnostic clarity. Beyond primary bone and soft tissue tumors, core biopsy also proves effective in accurately diagnosing bone infections such as osteomyelitis, thereby facilitating effective treatment strategies.
